# Cell viscoelasticity is linked to fluctuations in cell biomass distributions

**DOI:** 10.1038/s41598-020-64259-y

**Published:** 2020-05-04

**Authors:** Thang L. Nguyen, Edward R. Polanco, Alexander N. Patananan, Thomas A. Zangle, Michael A. Teitell

**Affiliations:** 1 Department of Bioengineering, University of California at Los Angeles, Los Angeles, CA 90095 USA; 2 Department of Chemical Engineering, University of Utah, Salt Lake City, UT 84112 USA; 3 Deparment of Pathology and Laboratory Medicine, University of California at Los Angeles, Los Angeles, CA 90095 USA; 4 Huntsman Cancer Institute, University of Utah, Salt Lake City, UT 84112 USA; 5 Molecular Biology Institute, University of California at Los Angeles, Los Angeles, CA 90095 USA; 6 Broad Center for Regenerative Medicine and Stem Cell Research, University of California at Los Angeles, Los Angeles, CA 90095 USA; 7 California NanoSystems Institute, University of California at Los Angeles, Los Angeles, CA 90095 USA; 8 Department of Pediatrics, University of California at Los Angeles, Los Angeles, CA 90095 USA; 9 Jonsson Comprehensive Cancer Center, David Geffen School of Medicine, University of California at Los Angeles, Los Angeles, CA 90095 USA

**Keywords:** Biophysical methods, Imaging, Microscopy

## Abstract

The viscoelastic properties of mammalian cells can vary with biological state, such as during the epithelial-to-mesenchymal (EMT) transition in cancer, and therefore may serve as a useful physical biomarker. To characterize stiffness, conventional techniques use cell contact or invasive probes and as a result are low throughput, labor intensive, and limited by probe placement. Here, we show that measurements of biomass fluctuations in cells using quantitative phase imaging (QPI) provides a probe-free, contact-free method for quantifying changes in cell viscoelasticity. In particular, QPI measurements reveal a characteristic underdamped response of changes in cell biomass distributions versus time. The effective stiffness and viscosity values extracted from these oscillations in cell biomass distributions correlate with effective cell stiffness and viscosity measured by atomic force microscopy (AFM). This result is consistent for multiple cell lines with varying degrees of cytoskeleton disruption and during the EMT. Overall, our study demonstrates that QPI can reproducibly quantify cell viscoelasticity.

## Introduction

Viscoelastic properties of cells are important emerging biomarkers of disease state and progression^1^. The simplest approach to defining cell viscoelastic properties examines two parameters: stiffness and viscosity, which characterize the elastic and dissipative components of a cell’s response to stress^2^. The elastic response has been used as a biomarker for cancer cells^3^ or metastatic potential^4^, and has been related to cell migration during embryogenesis^5^. Cell viscosity has been linked to multiple biological processes, such erythrocyte porous trafficking and deformability^6^, diffusion^7,8^, and cell disease state^9,10^.

Most approaches to interrogate cell viscoelastic properties use induced deformations^11^ or probes^12^. Approaches to measure the elastic component of cell viscoelasticity include atomic force microscopy (AFM)^13^, optical laser tweezers^14^, magnetic tweezers^15^, pipette suction^16^, uniaxial stretching rheometry^17^, hydrodynamic stretching^18^, and microrheology^19,20^. The viscous response of cells has been measured using approaches that include microrheology^19,20^, electronic spin resonance^21^, fluorescent rotor protein^22^, AFM^23^, pipette suction^16^, and optical laser tweezers^24^. These measurements, however, can be strongly influenced by the specific region of a cell that is probed^25^, alterations of the cytoskeletal network by an applied stress^26^ or cell interactions with a probe^27^. All of these influences may bias measurements of cell viscoelasticity.

Therefore, we developed a contact-free, non-invasive approach that accurately measures cell viscoelastic properties based on quantitative phase imaging (QPI), a method that we refer to as quantitative phase rheology (QPR). QPI^28^ is a microscopy technique used to measure the phase-shift or retardation of light due to its interactions with the relative dry mass, or the non-aqueous biomass, of a cell^29^. Using an experimentally determined cell-average specific refractive index, we can relate the phase shift of light to cell biomass^30,31^. QPI has been used to study cell growth^32^, death^33^, and responses to growth inhibition by chemotherapeutics or targeted inhibitors of biological processes^34–36^.

Previous studies have used what we refer to as QPR to measure membrane viscoelastic properties of enucleated erythrocytes, including development of an analytical model linking observed vibration modes to viscoelastic properties through the autocorrelation of quantitative phase data^37,38^. However, this model does not directly translate to the more complex structure of nucleated cells. In an application to nucleated cells, spatial and temporal autocorrelations of quantitative phase data from human pluripotent stem cell colonies indicated both a larger degree of spatial coordination and faster rate of temporal decorrelation for pluripotent cells compared to their differentiated progeny^39^. A more recent study found that spatial autocorrelations of quantitative phase data can be used to indicate the intracellular disorder of cells, a parameter related to cell stiffness in response to deformation to fluid shear^40^. Other work on QPR indicates that temporal autocorrelation of quantitative phase data relates to cellular transport properties including diffusion^41–43^, and show a correlative relation to cellular stiffness^44^. However, there is currently no QPR method to concurrently model and measure both the elastic and viscous components of cell viscoelasticity.

In the present study, we report that the temporal autocovariance of quantitative phase data for cells at interphase of the cell cycle show a response similar to a mass spring damper system. The elastic and viscous coefficients describing this behavior correlate with viscous and elastic stiffness components of interphase cells quantified by AFM measurements. We varied the cell stiffness of three different cell lines with cytochalasin B^45^, an actin polymerization inhibitor, and show a high correlation between QPR results and AFM viscoelasticity measurements. Finally, to validate our measurements in cells of the same genetic origin during a cell state transition in which stiffness plays a physiological role, we apply QPR to a cellular model of the epithelial to mesenchymal transition (EMT)^46^. These results show that QPR measures of stiffness and viscosity correlate with EMT state. Overall, our results suggest that label-free QPR can be used to indicate cell stiffness and viscosity, significantly expanding the utility of QPI for monitoring cell behavior.

## Results

### Autocovariance of cell QPI data exhibits damped oscillations

**Figure 1:**
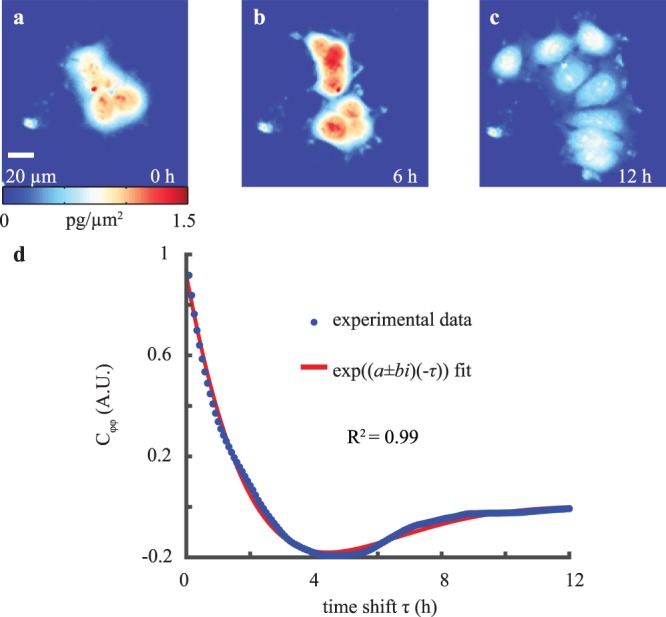
Autocovariance of QPI biomass-density over time displays underdamped oscillations. (**a***–***c**) QPI of MCF-7 cell cluster at 0, 6, and 12 h of imaging. (**d**) Autocovariance of QPI data over timeshift *τ* fitted to a complex exponential.

We used QPI to measure cellular biomass distribution over time (Fig. 1a–c) and computed the autocovariance of these biomass distributions over time, *C*_*ϕϕ*_, to quantify changes in the distribution of biomass caused by the motion of cellular structures (Fig. 1d). The autocovariance of the quantitative phase data (Fig. 1d) is well-fit by an equation describing damped harmonic oscillations (R^2^ = 0.99). The fitting coefficients in this equation are related to an effective stiffness, *k*, and effective viscosity, *μ*. Assuming the spring and damper act in series, *k* is given by Eq. (9) (Methods) and *μ* can be found by dividing Eq. (9) by Eq. (5) (Methods).

### Automated detection and removal of cell division events in quantitative phase data

QPR detects large changes in both effective stiffness and viscosity during mitosis (Fig. S1). These changes are consistent with previously measured increases in cortical tension and cell stiffness during cell division and mitosis^47–49^. However, our QPR analysis averages values obtained over a period of approximately 5 h, so changes in cell stiffness due to single mitotic events are not resolved. To measure population-level differences, we therefore restrict our analysis to interphase cells.

We filtered QPI data to automatically detect the localized increase in biomass density that occurs during mitosis using a kernel consisting of a sigmoid function in time^50^ and a disk in space. This kernel mimics the characteristic changes in cell phase shift that occur during mitotic cell rounding. When applied using an image processing filter (e.g. imfilter in Matlab), this kernel highlights regions of mitotic cells, without requiring any additional labels (Fig. S2A,B). To validate this method of automatically detecting mitosis, we used FUCCI green fluorescence to mark mitotic cells (Fig. S2c). We observed>80% overlap between fluorescently labeled mitotic cells and cells with high values of the QPI mitosis filter, indicating robust detection of mitosis. We then calculated true positive versus false positive rates for detection of images that contain a division event (Fig. S2d). This allowed us to determine a filter threshold that gives a true positive rate of >0.95.

We then applied our label-free QPI mitotic filter to our autocovariance analysis. We calculated autocovariance on all possible 5 h subsets of each cell cluster dataset. Any subset that was determined to contain images with a mitotic event were removed from the analysis. This automatic filtering eliminates cells in mitosis from QPI data to enable biomass-density decorrelation rate measurements for interphase cells only.

### QPR measurements of elasticity and viscosity

**Figure 2:**
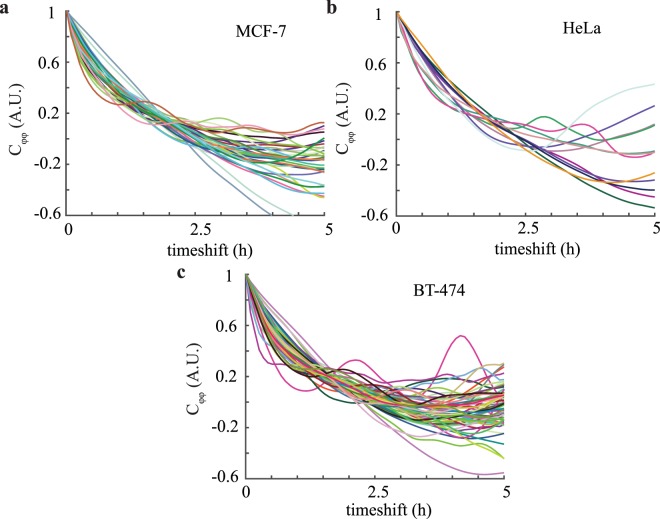
Autocovariance of QPI data from individual clusters and cells indicates significant heterogeneity. (**a**) Autocovariance of MCF-7 (*n* = 31), (**b**) HeLa (*n* = 12), and (**c**) BT-474 (*n* = 51) cells in 0 μM cytochalasin B with individual cell or cluster traces shown.

We performed QPR with filtered elimination of mitotic events for MCF-7 (Fig. 2a), HeLa (Fig. 2b), and BT-474 (Fig. 2c) cells. These curves display significant heterogeneity as detected by the variable periods and amplitudes of oscillation seen in the autocovariance curves of individual clusters. For example, BT-474 cells displayed the highest frequency of oscillation (*b* = 0.46 ± 0.07) and steepest exponential decay (*a* = 0.63 ± 0.05) (Fig. 2c) compared to the other two cell lines, whereas HeLa cells appear to have the lowest oscillation frequency and exponential decay (*b* = 0.24 ± 0.11 and *a* = 0.52 ± 0.12) (Fig. 2b). These qualitative differences correspond to a predicted lowest effective elasticity (aka stiffness) and viscosity for HeLa cells and a highest effective stiffness and viscosity for BT-474 cells. The standard deviation for stiffness from repeated measurements of single cells and clusters was 7–10%. The population standard deviation, however, was significantly larger, approximately 100%., indicating significant biological heterogeneity within each cell population.

**Figure 3:**
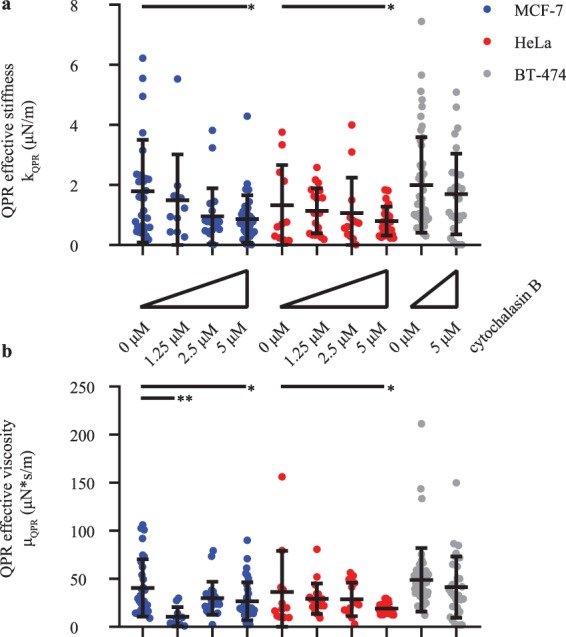
Population average QPR stiffness and viscosity values decrease with increasing cytochalasin B concentration. (**a**) QPR stiffness and (**b**) QPR viscosity of MCF-7, HeLa, and BT-474 over various 0-10 μM cytochalasin B concentration. QPR samples were collected at 0 μM (*n* = 12), 1.25 μM (*n* = 20), 2.5 μM (*n* = 14), and 5 μM (*n* = 25) for HeLa, at 0 μM (*n* = 31), 1.25 μM (*n* = 11), 2.5 μM (*n* = 22), and 5 μM (*n* = 34) for MCF-7, and at 0 μM (*n* = 51) and 5 μM (*n* = 31) for BT-474 cells. Error bars represent SD. * *p* < 0.05 and ** *p* < 0.01.

To induce a change in cellular stiffness and viscosity, we used the actin polymerization inhibitor cytochalasin B to disrupt the cell cytoskeleton over a drug concentration range of 0–5 μM^51^ and measured effective stiffness and viscosity with QPR (Fig. 3). These data display both significant cell-to-cell heterogeneity as well as the expected trend of decreasing stiffness and viscosity with increasing cytochalasin B concentrations. This is most easily detected in the population averaged autocovariance signal between control (R^2^ = 0.99 ± 0.01) and 5 μM (R^2^ = 0.99 ± 0.01) cytochalasin B treated MCF-7 cells (Fig. S3a), where the decay of the autocovariance for 5 μM treated cells is more rapid than the control, indicating a decrease in viscoelasticity. This result is consistent with similar data obtained using dynamic light scattering by others for the effect of lowered viscoelasticity on autocovariance values^52^. The stiffness change detected for HeLa (Fig. S3b) and BT-474 (Fig. S3c) cells was less dramatic under these cytochalasin B treatment conditions. Therefore, there are lower differences between the control (HeLa R^2^ = 0.98 ± 0.01, BT-474 R^2^ = 0.98 ± 0.01) and 5 μM perturbation autocovariance values (HeLa R^2^ = 0.99 ± 0.01, BT-474 R^2^ = 0.99 ± 0.01) for these cells than for MCF-7 cells. The individual autocovariance values for both control and 5 μM cytochalasin B treated cells fits the damped harmonic oscillation equations well, as quantified by average R^2^ > 0.98 for all perturbations in all 3 cell types.

**Figure 4:**
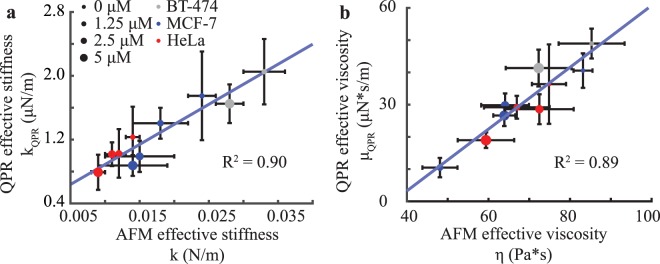
QPR predictions for stiffness and viscosity correlates with AFM data for multiple cell lines and drug concentrations. (**a**) QPR effective stiffness versus cell stiffness measured by AFM force curves for cells exposed to escalating doses of cytochalasin B. (**b**) QPR effective viscosity versus cell viscosity estimated with AFM by force dissipation. AFM data were collected at 0 μM (*n* = 75), 1.25 μM (*n* = 18), 2.5 μM (*n* = 37), and 5 μM (*n* = 133) for HeLa, at 0 μM (*n* = 72), 1.25 μM (*n* = 25), 2.5 μM (*n* = 20), and 5 μM (*n* = 66) for MCF-7, and at 0 μM (*n* = 12) and 5 μM (*n* = 28) for BT-474 cells. QPR samples were collected at the conditions and sample numbers indicated in Fig. 3. Error bars represent SEM.

We then compared QPR with AFM data as AFM is a validated ‘gold-standard’ method for measuring cell viscoelastic properties. We obtained a strong correlation (R^2^ = 0.9) between fit parameters for stiffness from QPR data compared with AFM measured stiffness values (Fig. 4a). QPR viscosity data also correlated well with AFM viscosity data with an R^2^ of 0.89 (Fig. 4b). Additionally, the material relaxation time (Fig. S4) computed from QPR measurements (Eq. (10), Methods) compares well to those of AFM relaxation of deformation under constant load from published studies^53,54^. Our measured values fall within the reported range^54,55^ for MCF-7 cells (23.2 ± 3.9 s), whereas the other cell types BT-474 (21.8 ± 4.1 s) and HeLa (38.0 ± 8.3 s) fall within the anticipated magnitudes for live cells, which ranges from seconds to one minute. These data indicate that QPR approaches provide reproducible and accurate label-free measurements of stiffness and viscosity.

### QPR measurements during EMT

**Figure 5:**
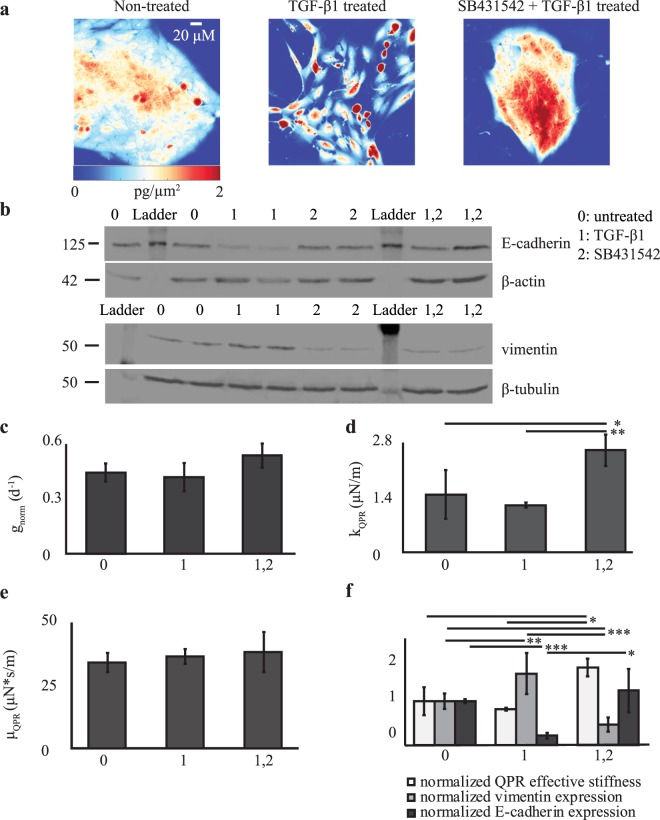
QPR quantifies changes in effective stiffness and viscosity during EMT. (**a**) Representative quantitative phase images of MCF-10A cells grown in control (non-treated) media, media supplemented with TGF-β1, and media supplemented with both TGF-β1 and SB431542. (**b**) Western blot of E-cadherin and vimentin expression in MCF-10A cells grown in untreated (control), TGF-β1, SB431542, or TGF-β1 + SB431542-containing media. β-actin and β-tubulin are loading controls, 2 independent biological replicates per sample. (**c**) Normalized growth rate, (**d**) QPR effective stiffness, and (**e**) QPR effective viscosity for MCF-10A cells grown in the listed conditions. (**f**) QPR stiffness, vimentin, and E-cadherin expression normalized to untreated cell values for MCF-10A cells grown in the listed conditions. Non-treated *n* = 20, TGF-β1 *n* = 41, and TGF-β1 + SB431542 *n* = 37. Error bars are SEM. * *p* < 0.05, ** *p* < 0.01, and *** *p* < 0.001.

We investigated whether QPR would be useful for measuring changes in effective stiffness and viscosity during changes in cell state. Therefore, we utilized our QPR approach for cells induced to undergo EMT. A shift from relatively stiff non-metastatic cancer cells to mechanically ‘softer’ cells with metastatic potential occurs during the EMT^56^, making this cell state transition an important model system. We induced EMT in MCF-10A cells by TGF-β1 exposure and observed profound morphological changes by QPI compared with control, untreated cells (Fig. 5a) that were consistent with previous studies^57^. MCF-10A cells exposed to the TGF-β receptor-inhibitor SB431542 also showed unique morphological features in QPI compared to untreated control and TGF-β1 treated cells (Fig. 5a). Reduced steady-state expression of the epithelial biomarker, E-cadherin, and increased expression of the mesenchymal biomarker, vimentin, in TGF-β1-treated cells confirmed a transition to a mesenchymal state (Fig. 5b). Conversely, SB431542-treatment enforced an epithelial state, confirmed by unchanged E-cadherin and markedly reduced vimentin steady-state expression levels (Fig. 5b). Untreated cells had intermediate levels of both proteins, suggesting a mixed population of cells in epithelial and mesenchymal states. Measurements of cell biomass from QPI showed no statistically significant differences in biomass accumulation rates between cells in these different biophysical states (Fig. 5c). However, a clear difference in QPR stiffness (*p*-value <0.05) (Fig. 5d) but not in viscosity (Fig. 5e), was obtained between SB431542-treated epithelial cells relative to untreated, mixed population, and TGF-β1-treated mesenchymal cells (Fig. 5a). Furthermore, an increase in E-cadherin expression in TGF-β1-treated cells corresponded with an increased stiffness that negatively correlates with vimentin expression (Fig. 5f). Overall, the data show that QPR stiffness could be an alternative, label-free physical biomarker for distinguishing cells in an epithelial state from those in a mesenchymal state, as well as cells comprising a mixed heterogeneous population.

## Discussion

Movement of cell biomass, quantified as the autocovariance of quantitative phase imaging measurements, displays harmonic oscillatory motion (Fig. 2). A two-parameter viscoelastic model captures the oscillation and decay of this autocovariance (Fig. 1d). Fitting this model to experimental data enables the extraction of separate values of effective stiffness and viscosity of a cell (Fig. 3). Although there are previous methods to measure stiffness^40^ with QPI data, our analysis method based on temporal measurements of cell biomass motion captures both stiffness and viscosity components of cell rheological properties. We refer to the measurement of these and other^40–43^ rheological properties of a cell using QPI as QPR.

To use our QPR measurements to distinguish between different cell types, states and conditions, we assume a consistent stiffness over the measurement period of approximately 5 h. This assumption is not applicable during mitosis in which cell stiffness changes dramatically^49^. We therefore developed an automated method to detect cell divisions for their removal from quantitative phase imaging data. This enables QPR to automatically process live cell QPI data and extract cell rheological properties. Future work could examine improvements to the spatial and temporal resolution of QPR required to capture the magnitude of cell viscoelastic changes during mitosis.

We observed a strong correlation between QPR measurements of cell stiffness (Fig. 4a) and viscosity (Fig. 4b) relative to AFM measurements. Relaxation times computed from QPR data are also within the same range observed previously with AFM (Fig. S4)^54,55^. This suggests that QPR measures cell viscoelasticity within a similar force and time regime as AFM measurements.

A physical interpretation of our results can be found in the model proposed by Qian^58^ for single particle tracking within a Kelvin-Voight material. This model gives similar predictions to the series spring damper (Maxwell) material model we apply, suggesting that QPR is effectively tracking displacements of small particles of cell biomass immersed in a Maxwell material. When we applied the Kelvin-Voight model to our QPR data, we obtain a moderate fit to AFM viscosity values (R^2^ = 0.81, Fig. S5) compared to the fit for a Maxwell model (R^2^ = 0.89, Fig. 4b). This indicates that a Maxwell material model is the more appropriate two-parameter, linear viscoelastic material model for interpreting QPR data. Although this two-parameter, linear model represents a simplistic view of cell viscoelasticity, this model nonetheless captures the essential features recorded in our data.

We note that this physical interpretation of the mathematical model includes an inertia term, despite describing the behavior of a low Reynolds number fluid. We keep this term, which arises in models of underdamped systems^58^, to capture our observation of underdamped motion of cell biomass (Figs. 1d and 2). This phenomenological assumption, rooted in observation, allows us to fit a two-parameter viscoelastic model and extract cell rheological properties from QPI data that correlate to AFM values. In terms of a potential physical meaning of this term, recent work indicates that inertia-like oscillations can occur in actively driven, viscoelastic fluids^59^. As the cell is an active material^60^, we speculate that the inertia-like behavior we record in our system is due to a similar coupling between viscoelastic material properties and active force generation from cytoskeletal rearrangements. This suggests the need for future modeling based on a more sophisticated cell material model that can better incorporate these cellular mechanics.

Despite the correlation between AFM and QPR measurements of stiffness and viscosity, there is a large difference in magnitude of these values. This difference is partially explained by differences in probe size. The radius of the AFM probe tip is 500 nm, whereas the effective probe for QPR is the material within the cytoplasm. The observed difference in magnitude of QPR stiffness relative to AFM stiffness is ~10^4^, suggesting a QPR probe size of ~5 nm. This probe size is within the range for a complex of average sized proteins that constitute the majority of mammalian cell biomass. For example, in eukaryotes a ‘typical’ ~3 nm in radius^61^ protein has an average biomass of ~56 kDa^62^. The difference in magnitude of AFM and QPR measurements can therefore be explained by the difference in the cross sectional area of these probes. Furthermore, we model the cell as a purely linear viscoelastic material; however, in general, cell rheology is dependent on length scale, strain rate, and magnitude of applied force which may differ between these two approaches. In addition, AFM measures viscosity from viscous dissipation, whereas QPR measures an effective frictional coefficient felt by a particle due to the viscosity of a cell. These are technically two different properties that are closely related through viscosity. Our QPR method is most similar to passive particle tracking in microrheology^63^, which provides a stiffness value from the expected relationship to passive particle motion. Microrheology measurements^12^ when compared to AFM measurements^64^ for mouse embryonic fibroblasts show large differences in measured magnitudes: 14 Pa for microrheology stiffness versus 7.7 kPa for AFM stiffness. A similar order of magnitude difference between AFM stiffness and microrheology stiffness was also obtained for MCF10A breast epithelial cells as well^65^.

Additional parameters that may affect QPR measurements include the frequency of measurements^66^ and the ratio of water content to cell volume of our samples^67,68^. To interrogate the effect of measurement frequency, we obtained QPR viscosity and elastic modulus data over a range of measurement frequencies for a single MCF-7 cell cluster and for a population of cells (Fig. S6). We observed that both stiffness and viscosity values are within the standard error of the mean (SEM) for QPI measurements at frequencies less than 15 min per frame. However, for QPI measurement frequencies above ~30 min per frame, measurement accuracy and stability begins to deviate from the SEM. For water content effects, cells persist within a physiological range of 260–320 mOSM/kg^69^, or within a range of ~60 mM for osmolality, with water losses of 10–15% or less^70^. Mechanisms^71^ that maintain this homeostasis are tightly controlled and regulating both osmolality and water content losses. These values indicate, for the physiologically-relevant cell culture systems employed here, minimal osmolality or water content influences on QPR measurements. In addition, others have shown that significant stiffness changes require large changes in osmolality^68^ of at least 150 mM or in water content to change cell volumes^67^.

Overall, our results show the potential of a label-free and non-contact method that can measure cell rheological properties. As a transmitted light microscopy method, QPI is non-invasive and therefore minimizes the confounding effects of probes when examining biological processes in live cells. Because QPR builds on an existing quantitative phase imaging workflow, QPR can be integrated with other measurements already commonplace with quantitative phase techniques, such as cell biomass or biomass accumulation rate (Fig. 5c). This and previous studies^40–43,72^ on alternative approaches to QPR suggests that the use of quantitative phase imaging data to measure cell structure^39,40,72^ and how cell structure changes over time^41–44^ provides powerful methods in biophysical research of cell state and state transitions.

## Methods

### Cells and cell culture

MCF-7 and BT-474 human invasive ductal breast adenocarcinoma cells and MCF-10A immortalized human breast epithelial cells were purchased from the American Type Culture Collection (ATCC). HeLa human cervical adenocarcinoma cells expressing fluorescence ubiquitination cell cycle indicator (FUCCI)^73^ plasmids were received from Dr. Ran Kafri (University of Toronto). FUCCI plasmids include mKO2-hCdt1, a monomeric fast-folding variant of Kusabira Orange fused to amino acids 30–120 of human Cdt1, and mAG-hGem, a monomeric version of Azami green fused to amino acids 1–100 of human Geminin^73^. MCF-7 cells were also transiently transfected with FUCCI mKO2-hCdt1 and mAG-hGem expression plasmids using the BacMam system (Fisher). We cultured MCF-7 cells in EMEM supplemented with 10% fetal bovine serum (FBS, Omega Scientific) and 10 mg/L human recombinant insulin (Sigma). BT-474 cells grew in Hybri-Care Medium (ATCC) reconstituted in cell culture grade water (Fisher) with 1.5 g/L sodium bicarbonate and 10% FBS (Omega Scientific). MCF-10A cells grew in MEGM Bulletkit media (Lonza) with cholera toxin (Sigma-Aldrich) at 100 ng/mL and without gentamycin-amphotericin B mix. HeLa cells were cultured in DMEM with 4.5 g/L glucose, L-glutamine, and sodium pyruvate (Cellgro) along with 1% penicillin streptomycin (Cellgro), 1% Q-max (Gibco), 1% non-essential amino acids (Gibco), and 10% FBS (Omega Scientific). We incubated cells with escalating doses of cytochalasin B (Sigma Aldrich) dissolved in DMSO solution or to 0.1% DMSO control, starting 4 h prior to experiments.

### Quantitative phase and fluorescence imaging

QPI of MCF-7, BT-474, and HeLa cells was performed as described in Yu *et al*.^74^. Fluorescence images were obtained with an EM-CCD C9100 camera (Hamamatsu Photonics) and an X-Cite Series 120 Q (Lumen Dynamics) source. Image collection occurred every 5 min for 12 h at 14–16 imaging locations containing cells plated with sufficient spacing to enable automated image processing and biomass segmentation.

### Quantitative phase image analysis

Image processing was performed using custom MATLAB (MathWorks) scripts. Cells and cell clusters were identified and segmented using a local adaptive threshold based on Otsu’s method^39,75^ and particle tracking code based on Grier *et al*.^50,76^. Compensation for translational motion was done by finding the maximum two-dimensional cross correlation of each cell or cell cluster image against the immediately prior image. Manual detection of interphase, mitotic, and cell division boundaries was by visual inspection of image frames containing cells whose mean phase-shift increased, followed by splitting into two daughter cells then a decrease in mean phase-shift. Automated detection of an interphase-mitotic event boundary was by pattern matching biomass distribution images with a mitotic filter consisting of a one-dimensional sigmoid filter in time^77^ and a two-dimensional disk filter of diameter 5 pixels in space. A mitotic filter value threshold of 0.6 A.U. for MCF-7, BT-474, and HeLa cells was chosen to maximize the true positive and minimize the false positive rates for entry into mitosis by validation with manual detection and fluorescence data.

### AFM

AFM experiments were performed on a Bioscope Resolve BioAFM using a MLCT D triangular probe tip at 37 °C (Bruker). Spring constants of cantilever tips measured 0.03–0.08 N/m and were calibrated with nanoscope measurement acquisition software (Bruker). Samples were incubated in media containing 0.1% DMSO or escalating doses of cytochalasin B for 4 h prior to the experiment with an additional 30 min of system equilibration with the cantilever submerged. The cantilever was calibrated using Nanoscope measurement acquisition software (Bruker). We analyzed force curves by finding the slope of the linear region of the curve measured during cantilever retraction of interphase cells in order to eliminate artifacts from pushing cells against the culture plate. This corresponded to the region from 20% to 80% of the maximum applied force on the cell (Fig. S7). Viscosity measurements were extracted from force curve data by calculating the area between the extended and retraction force curves as performed in Rebelo *et al*.^23^ (Fig. S7, shaded region).

### Biomass accumulation rate calculation

Quantitative phase biomass distribution images were summed over the projected area of each cell cluster to obtain the total biomass per cluster at specified time points. We calculated exponential biomass accumulation rates by taking the logarithm of the biomass over time data and fitting to a first order polynomial equation using MATLAB Polyfit (MathWorks).

### EMT

One day before EMT induction, MCF-10A cells were placed in standard 6-well culture plates. Recombinant human TGF-β1 (Sigma-Aldrich) was added to the culture media at 5 ng/mL to induce EMT. Alternatively, the TGF-β receptor inhibitor SB-431542 (Sigma-Aldrich) was added to the culture media at 10 μM final concentration to enforce an epithelial phenotype on MCF-10A cells. Cell exposure to these conditions for 7 d ensured full effects^46^. Cells re-plated for imaging or Western blot studies were cultured with no additives, 5 ng/mL TGF-β1, 10 μM SB-431532, or both agents together. We incubated cells with or without additives for 2 d before imaging or Western blot.

### Western blot

MCF-10A cells were harvested and lysed in 2 mL of ice cold sample buffer containing 7 mL of 0.5 Tris-HCl (Sigma-Aldrich), 3 mL glycerol (Sigma-Aldrich), and 1 g of sodium dodecyl sulfate (SDS) (Sigma-Aldrich) mixed with 1.2 mg of bromophenol blue (Sigma-Aldrich). 30 ug of protein lysates with 3 μL β-mercaptoethanol (Sigma-Aldrich) were loaded on a 10% polyacrylamide gel (Sigma-Aldrich), electrophoresed, and then transferred to a nitrocellulose membrane (Fisher). Membranes were incubated overnight with primary antibodies against β-actin (Sigma-Aldrich, A2066), E-cadherin (Cell Signaling Technology, 14472 s), β-tubulin (R&D Systems, MAB1195), or vimentin (Cell Signaling Technology, 5741 s). This was followed by incubation for 2 h with a secondary antibody solution containing Li-Cor TBS blocking solution (Li-Cor) and either IRDye 800CW goat anti-rabbit (Li-Cor, 926–32211) and IRDye 680RD donkey anti-mouse (Li-Cor, 926–68072) or IRDye 800CW goat anti-mouse (Li-Cor, 926–68070) or IRDye 680RD donkey anti-rabbit (Li-Cor, 926–32214) antibodies and then imaging on a Li-Cor Odyssey FC (Li-Cor). Protein abundance was normalized to either β-tubulin or β-actin for quantification of western blot data.

### Statistical analysis

Statistical analyses were performed using two-tailed Student’s t-test with unequal variances and sample size (Welch’s t-test).

### Autocovariance calculation from quantitative phase data

To measure the similarity of quantitative phase data over time we used an unbiased estimate of autocovariance^78^ of the phase-shift signal, which is an autocorrelation of the mean subtracted data. We normalized the temporal autocovariance to the number of data points used in each autocovariance window, referenced to the end of the time shift window (*t*_0_), and defined as:

1 $${C}_{\phi \phi }(x,y,{t}_{0},\tau )=\frac{w\mathop{\sum }\limits_{j=0}^{w-\tau /\varDelta t}(\phi (x,y,{t}_{0}-j\varDelta t)-\langle \phi (x,y,{t}_{0})\rangle )\cdot (\phi (x,y,{t}_{0}-j\varDelta t-\tau )-\langle \phi (x,y,{t}_{0})\rangle )}{(w-\frac{\tau }{\varDelta t})\mathop{\sum }\limits_{j=0}^{w-\tau /\varDelta t}{(\phi (x,y,{t}_{0}-j\varDelta t)-\langle \phi (x,y,{t}_{0})\rangle )}^{2}}$$

where *x* and *y* are position after removing rigid translational motion of the cell cluster, *t*_0_ is the initial time or time of the first position in the time window, *ϕ* is phase shift, *N* is the number of data points used to calculate the signal, *w* is the number of images, Δ*t* is time between measurements, and *τ* is time shift. The autocovariance was then averaged over a cell or cell cluster area as:

2 $${\langle {C}_{\phi \phi }({t}_{0},\tau )\rangle }_{x,y}=\frac{1}{A}\sum _{{\rm{all}}x,y{\rm{in}}A}{C}_{\phi \phi }(x,y,{t}_{0},\tau )$$

where *A* is the area of a cell or cell cluster in pixels. We also took the average of the autocovariance through time for all times corresponding to interphase cells,

3 $${\langle {C}_{\phi \phi }(\tau )\rangle }_{x,y,t}=\frac{1}{n}\sum _{{\rm{allinterphase}}{t}_{0}}{\langle {C}_{\phi \phi }({t}_{0},\tau )\rangle }_{x,y}$$

where *n* is the number of different end time points.

### Predicted autocovariance of cell biomass distributions

Using biomass as a tracer for displacement and translating this equation into autocovariance space yields:

4 $${\langle {C}_{\phi \phi }(\tau )\rangle }_{x,y,t}=\left(\frac{w}{w-\frac{\tau }{\varDelta t}}\right)\left(1-\frac{\mathop{\sum }\limits_{j=w-\tau /\varDelta t}^{w}\phi (j\varDelta t)\cdot \phi (j\varDelta t)}{\mathop{\sum }\limits_{j=0}^{w}\phi (j\varDelta t)\cdot \phi (j\varDelta t)}\right)\exp ((a\pm b\omega i)(-\tau )).$$

If we assume the observed damped oscillations are due to a series, harmonic *a* and *b* can be written as:

5 $$a=\frac{k}{2\mu }$$

6 $$b={\left(\frac{k}{\langle m\rangle }\right)}^{1/2}{\left(1-\frac{k\langle m\rangle }{4{\mu }^{2}}\right)}^{1/2}$$

where *k* is the effective spring constant of the cell felt by the particle over the measurement period, *μ* is the effective damping coefficient from the viscous forces of the cell felt by the particle, and $\langle m\rangle$ is the average biomass of particles in our system. Assuming that the system is ergodic,

7 $${\langle \phi (j\varDelta t)\rangle }_{w-\tau /\varDelta t}^{w}\approx {\langle \phi (j\varDelta t)\rangle }_{0}^{w}$$

the autocovariance equation then reduces to:

8 $${\langle {C}_{\phi \phi }(\tau )\rangle }_{x,y,t}=\exp ((a\pm bi)(\,-\,\tau )).$$

This means that effective stiffness can be described as:

9 $$\frac{k}{\langle m\rangle }={a}^{2}+{b}^{2}$$

and the effective viscosity can be found from dividing Eq. (9) by Eq. (5). Relaxation time *t*_*relax*_ was calculated as:

10 $${t}_{relax}=\frac{1}{2a}\varDelta t$$

where *Δt* is the time interval between measurements.

## Supplementary information

Supplementary Information.
