# Supplementary material for: Cell viscoelasticity is linked to fluctuations in cell biomass distributions
Source: Sci Rep. 2020 May 4;10:7403. doi: 10.1038/s41598-020-64259-y (PMC7198624; doi:10.1038/s41598-020-64259-y)
Supplement: Supplementary file 1 — Supplementary Information. [file 41598_2020_64259_MOESM1_ESM.pdf]

Cell viscoelasticity is linked to fluctuations in cell biomass distributions

Thang L. Nguyen, Edward R. Polanco, Alexander N. Patananan, Thomas A. Zangle, and Michael A. Teitell

SUPPLEMENTARY INFORMATION

Supplementary Derivation

Supplementary Figures S1 – S7

Supplementary Unprocessed Western Blots (related to Fig. 5B)

Supplementary References

SUPPLEMENTARY DERIVATION

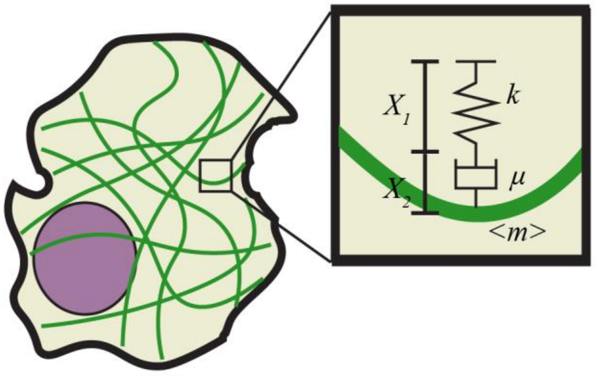

Summary:

We provide a derivation for how quantitative phase rheology (QPR) generates effective cell stiffness and viscosity using the autovariance of quantitative phase imaging (QPI) data. To do this, we establish a basic definition of the autovariance function and a basic equation that extracts stiffness and viscosity from dry mass, or non-aqueous biomass, displacement. We combine this definition and equation with QPI data to extract stiffness and viscosity regimes from the autovariance function.

Autocovariance

To measure the similarity of quantitative phase data over time we used an unbiased estimate of autocovariance[1] of the phase-shift signal, which is an autocorrelation of the mean subtracted data. We normalized the temporal autocovariance to the number of data points used in each autocovariance window, referenced to the end of the time shift window (t_0), and defined as:

$$C_{\phi\phi}(x,y,t_0,\tau)=\frac{w\sum_{j=0}^{w-\tau/\Delta t}(\phi(x,y,t_0-j\Delta t)-\langle\phi(x,y,t_0)\rangle)\cdot(\phi(x,y,t_0-j\Delta t-\tau)-\langle\phi(x,y,t_0)\rangle)}{(w-\frac{\tau}{\Delta t})\sum_{j=0}^{w-\tau/\Delta t}(\phi(x,y,t_0-j\Delta t)-\langle\phi(x,y,t_0)\rangle)^2} \quad \text{(S1)}$$

Where x and y are positions after removing rigid translational motion of a cell cluster, t_0 is the time, ϕ is phase-shift, N is the number of data points used to calculate the signal, w is the number of images, Δt is time between measurements, and τ is time lag. The autocovariance was then averaged over a cell or cell cluster area as:

$$\langle C_{\phi\phi}(t_0,\tau)\rangle_{x,y}=\frac{1}{A}\sum_{\text{all }x,y\text{ in }A}C_{\phi\phi}(x,y,t_0,\tau) \quad \text{(S2)}$$

where A is the area of a cell or cell cluster in imaging pixels. We also took the average of the autocovariance through time for all times corresponding to cells in interphase of the cell cycle,

$$\langle C_{\phi\phi}(\tau)\rangle_{x,y,t}=\frac{1}{n}\sum_{\text{all interphase }t_0}\langle C_{\phi\phi}(t_0,\tau)\rangle_{x,y} \quad \text{(S3)}$$

where n is the number of different end time points.

Two-parameter

We treat the cellular structures imaged by quantitative phase as particles immersed in a Maxwell liquid (Fig. 1A). Therefore, these structures feel the effect of a spring damper system in series described as the following system of equations:

$$\langle m\rangle \frac{d^2 X_{tot}}{dt^2} = -\mu \frac{dX_2}{dt} + f(t) \tag{S4}$$

$$kX_1 = \mu \frac{dX_2}{dt} \tag{S5}$$

$$X_{tot} = X_1 + X_2 \tag{S6}$$

where k is the long term effective spring constant of the cell felt by a particle, μ is the effective damping coefficient from the viscous forces of the cell felt by a particle, $f(t)$ is the applied impulse force, X_1 is the elastic displacement, X_2 is the viscous displacement, X_{tot} is the total displacement of the biomass, and $\langle m\rangle$ is the average biomass of particles in the system. We observe long timescales that are much greater than the average relaxation times of a cell (Fig. S3), so the long timescale effects dominate and the active force can be considered as applied nearly instantaneously. Rearranging equation (S4) in terms of only the elastic displacement X_1 or only the viscous displacement X_2 yields the following differential equations:

$$\langle m\rangle \frac{d^2 X_1}{dt^2} = -kX_1 - \frac{k\langle m\rangle}{\mu}\frac{dX_1}{dt} \tag{S7}$$

$$\langle m\rangle \frac{d^3 X_2}{dt^3} = -k\frac{dX_2}{dt} - \frac{k\langle m\rangle}{\mu}\frac{d^2 X_2}{dt^2} \quad . \tag{S8}$$

Assuming that the total displacement X_{tot} contributes to the majority of biomass rearrangement and oscillation, we integrate equation (S8) over time, add it to equation (S7), and rearrange this equation of a damper spring system in series to obtain an inhomogeneous ordinary differential equation (ODE) for the total displacement:

$$\langle m\rangle \frac{d^2 X_{tot}}{dt^2} = -kX_{tot} - \frac{k\langle m\rangle}{\mu}\frac{dX_{tot}}{dt} + c_1 \tag{S9}$$

where c_1 could be seen as a buildup of stress from past deformation or a memory function. Solving for the general solution of equation (S9) gives a homogenous component and a particular solution by the method of undetermined coefficients:

$$X_{tot,\mathrm{h}}(t) = \left(X_{tot,0} - X_{tot,rest}\right)\exp\left(\left(\frac{k}{2\mu} \pm \left(\frac{k}{\langle m\rangle}\right)^{1/2}\left(1 - \frac{k\langle m\rangle}{4\mu^2}\right)^{1/2} i\right)t\right) \quad \text{(S10)}$$

$$X_{tot,p}(t) = \frac{c_1}{k} \equiv X_{tot,rest} \quad \text{(S11)}$$

where $X_{tot,0}$ is the initial displacement and $X_{tot,rest}$ is the long term resting displacement of our system. Because the relaxation timescale (Fig. S3) is over an order of magnitude lower than the period of measurement, the active force can be modeled as an instantaneous displacement represented as a delta function, δ, at some time, t_j, not equal to zero. Solution of this spring damper system without this active force yields:

$$X_{tot}(t) = \left(X_{tot,0} - X_{tot,rest}\right)\exp\left(\left(\frac{k}{2\mu} \pm \left(\frac{k}{\langle m\rangle}\right)^{1/2}\left(1 - \frac{k\langle m\rangle}{4\mu^2}\right)^{1/2} i\right)t\right) + X_{tot,rest}\ . \quad \text{(S12)}$$

With each individual impulse the displacement from the active force can be modeled as:

$$X_{tot}(t) = \left(X_{tot,0} - X_{tot,rest} + \delta(t - t_j)\right)\exp\left(\left(\frac{k}{2\mu} \pm \left(\frac{k}{\langle m\rangle}\right)^{1/2}\left(1 - \frac{k\langle m\rangle}{4\mu^2}\right)^{1/2} i\right)t\right) + X_{tot,rest} \quad \text{(S13)}$$

where t_j is the time of each impulse displacement. Assuming a linear viscoelastic material, the total displacement in time can be represented as the superposition of the various impulse displacements from the active forces, which then simplifies to:

$$X_{tot}(t) = \left(X_{tot,0} - X_{tot,rest} + \sum_{t_j \neq 0}^{T} \delta(t - t_j)\right)\exp\left(\left(\frac{k}{2\mu} \pm \left(\frac{k}{\langle m\rangle}\right)^{1/2}\left(1 - \frac{k\langle m\rangle}{4\mu^2}\right)^{1/2} i\right)t\right) + X_{tot,rest} \quad \text{(S14)}$$

where T is the period of observation. We then establish the relationship between biomass and displacement of biomass by:

$$\frac{DM(\vec{x},t)}{Dt} = \frac{\partial M(\vec{x},t)}{\partial t} + \vec{v}(\vec{x},t) \bullet \nabla M(\vec{x},t) \tag{S15}$$

where M is biomass as a function of position x and time t, and v is velocity as a function of x and t. We assume that the main contribution to the partial derivative of biomass with time is due to growth and since our measurement occurs over a short time interval, growth is negligible, therefore:

$$\frac{DM(\vec{x},t)}{Dt} = \vec{v}(\vec{x},t) \bullet \nabla M(\vec{x},t) \ . \tag{S16}$$

We further assume that the cell velocity, v, and biomass, M, fields are isotropic with no dependence on direction. Averaging over θ in polar coordinates yields:

$$\left\langle \frac{DM(r,t)}{Dt} \right\rangle_\theta = \left\langle v(r,t) \right\rangle_\theta \left(\frac{1}{r} \frac{\partial \left(r \left\langle M(r,t) \right\rangle_\theta \right)}{\partial r} \right) = \left\langle v(r,t) \right\rangle_\theta \left(\frac{1}{r} \left\langle M(r,t) \right\rangle_\theta + \frac{\partial \left\langle M(r,t) \right\rangle_\theta}{\partial r} \right). \tag{S17}$$

Assuming that this change in biomass over radial distance is small compared to the total biomass over a radial distance r we obtain:

$$\left\langle \frac{DM(r,t)}{Dt} \right\rangle_\theta = \left\langle v(r,t) \right\rangle_\theta \left(\frac{\left\langle M(r,t) \right\rangle_\theta}{r} \right) \ . \tag{S18}$$

Averaging over a radial distance and assuming that velocity, v, radial position, r, and biomass, M, do not correlate over the radial distance because the system is isotropic we obtain:

$$\left\langle \frac{DM(t)}{Dt} \right\rangle_{r,\theta} = \left\langle \left\langle v(r,t) \right\rangle_\theta \frac{\left\langle M(r,t) \right\rangle_\theta}{r} \right\rangle_r = \left\langle v(t) \right\rangle_{r,\theta} \left(\frac{\left\langle M(t) \right\rangle_{r,\theta}}{\left\langle r \right\rangle} \right) \ . \tag{S19}$$

Assuming this system is ergodic, the local spatial average of biomass is equal to the temporal average biomass, which is constant with respect to time, and therefore the average biomass over radial distance term is only a function of *r*, which we call $\kappa(r)$:

$$\kappa(r) \equiv \frac{\gamma(r)}{\langle r \rangle} \approx \frac{\langle M(t) \rangle_{r,\theta}}{\langle r \rangle} \tag{S20}$$

where $\gamma(r)$ is the local spatial average of the biomass, which is constant over time and is therefore only a function of radial position:

$$\left\langle \frac{DM(t)}{Dt} \right\rangle_{r,\theta} = \kappa(r) \langle v(t) \rangle_{r,\theta} = \kappa(r) \left\langle \frac{DX(t)}{Dt} \right\rangle_{r,\vartheta} . \tag{S21}$$

Since κ is independent of time, we can integrate and obtain:

$$\langle M(t) \rangle_{r,\theta} = \kappa(r) \langle X(t) \rangle_{r,\theta} . \tag{S22}$$

Thus, the ratio of biomass over initial biomass is equivalent to the displacement over initial displacement:

$$\frac{\langle M(t) \rangle_{r,\theta}}{\langle M(t_0) \rangle_{r,\theta}} = \frac{\kappa(r) \langle X(t) \rangle_{r,\theta}}{\kappa(r) \langle X(t_0) \rangle_{r,\theta}} = \frac{\langle X(t) \rangle_{r,\theta}}{\langle X(t_0) \rangle_{r,\theta}} . \tag{S23}$$

The biomass for a particular area is directly proportional to the phase-shift[4-7]:

$$M(\vec{x},t) = 1/\alpha \cdot \phi(\vec{x},t) \tag{S24}$$

where ϕ is phase-shift, and α is the specific refractive index, which is determined experimentally. Therefore, phase-shift data, ϕ, obtained via QPI can be used to obtain information about the displacement of cell biomass over time.

Predicted autocovariance of cell biomass distributions

Using biomass as a tracer for displacement and translating this equation into autocovariance space yields:

$$\left\langle C_{\phi\phi}(\tau)\right\rangle_{x,y,t} = \left(\frac{w}{w-\dfrac{\tau}{\Delta t}}\right)\left(1-\frac{\sum_{j=w-\tau/\Delta t}^{w}\phi(j\Delta t)\cdot\phi(j\Delta t)}{\sum_{j=0}^{w}\phi(j\Delta t)\cdot\phi(j\Delta t)}\right)\exp\left(\left(a\pm b\omega i\right)\left(-\tau\right)\right) \qquad \text{(S25)}$$

where a and b are described in terms of coefficients as:

$$a=\frac{k}{2\mu} \qquad \text{(S26)}$$

$$b=\left(\frac{k}{\langle m\rangle}\right)^{1/2}\left(1-\frac{k\langle m\rangle}{4\mu^2}\right)^{1/2} \qquad \text{(S27)}$$

where w, τ, ϕ, and Δt are the same as in equation (S12), the average autocovariance function is the same as in equation (S3), and a and b are the coefficients described in equations (S26) and (S27). Assuming that the system is ergodic:

$$\left\langle\phi(j\Delta t)\right\rangle_{w-\tau/\Delta t}^{w}\approx\left\langle\phi(j\Delta t)\right\rangle_{0}^{w} \qquad \text{(S28)}$$

$$\left\langle\phi(j\Delta t)\right\rangle_{w-\tau/\Delta t}^{w}\cdot\left\langle\phi(j\Delta t)\right\rangle_{w-\tau/\Delta t}^{w}\approx\left\langle\phi(j\Delta t)\right\rangle_{0}^{w}\cdot\left\langle\phi(j\Delta t)\right\rangle_{0}^{w}=c\left\langle\phi(j\Delta t)\right\rangle^{2} \qquad \text{(S29)}$$

where

$$c=\frac{\sum_{j=0}^{w}\phi(j\Delta t)^2-w\left\langle\phi(j\Delta t)\right\rangle^{2}}{w}\,. \qquad \text{(S30)}$$

The autocovariance equation then reduces to:

$$\left\langle C_{\phi\phi}(\tau)\right\rangle_{x,y,t}=\exp((a\pm bi)(-\tau))\,. \qquad \text{(S31)}$$

This means that the fitting parameter, a, describes the effective damping particles encounter within the cell, whereas the effective stiffness is described as:

$$\frac{k}{\langle m \rangle} = a^2 + b^2 . \quad \text{(S32)}$$

Relaxation time, τ_{relax}, was calculated as:

$$\tau_{relax} = \frac{1}{2a} \Delta t \quad \text{(S33)}$$

where Δt is the time interval between measurements.

SUPPLEMENTARY FIGURES

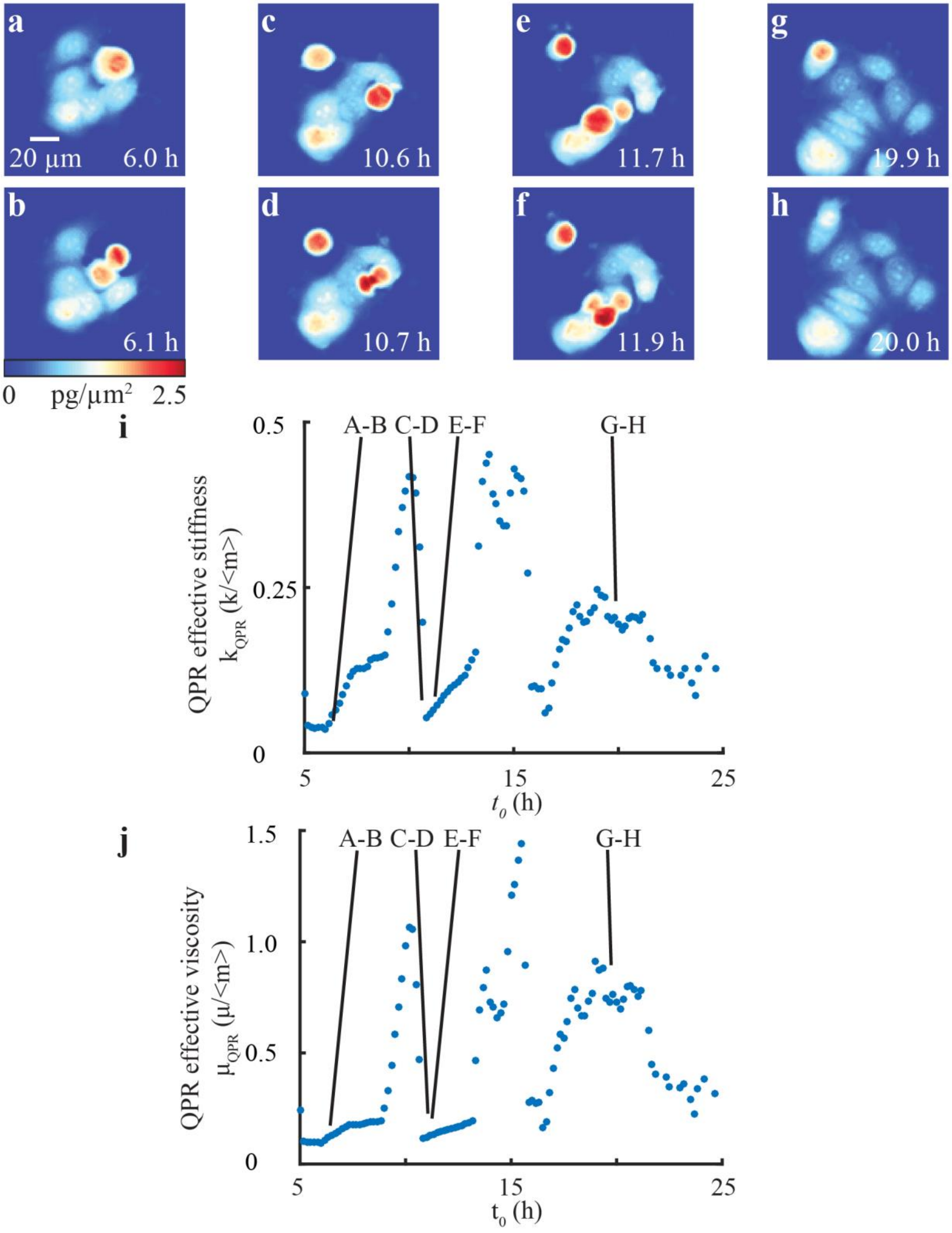

FIGURE S1 Cell division induces large mass fluctuations affecting QPR stiffness and viscosity measurements. (**a**, **c**, **e**, **g**) QPI of MCF-7 colonies before each mitosis plotted as 4 decorrelation rate peaks in (**i**). (**b**, **c**, **f**, **h**) QPI of MCF-7 colonies after each mitosis corresponding to the period after each trough in (**i**). (**i**) Time course of QPR stiffness measurement for MCF-7 cell colony in (**a**-**h**) with 4 cell divisions during 24 h of QPI. (**j**) Time course of QPR viscosity measurement for MCF-7 cell colony in (**a**-**h**) with 4 cell divisions over 24 h of QPI.

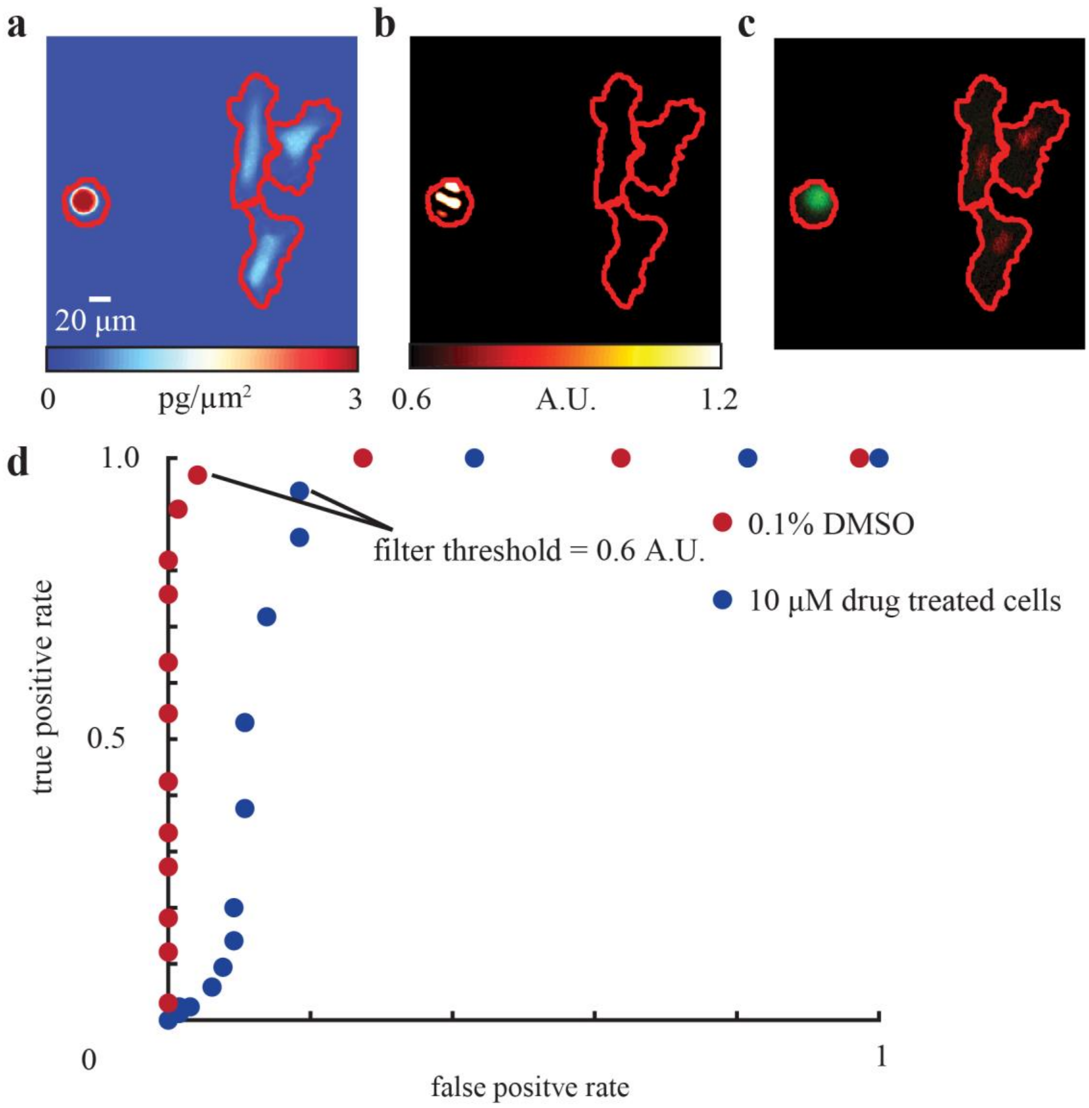

FIGURE S2 Assessment of cell division by fluorescence and QPI to remove mitotic events. (**a**) QPI of MCF-7 cells. (**b**) Filtered image of the same MCF-7 cells in (**b**) used to identify mitosis and cell division. The filter kernel consists of a sigmoid function in time and a disk in space to mimic and highlight round cells with a large phase shift in mitosis. (**c**) Simultaneous fluorescence image of MCF-7 cells in (**b** and **c**) expressing FUCCI fluorescence ubiquitination cell cycle indicator plasmids mKO2-hCdt1 and mAG-hGem. (**d**) ROC curves for detecting

MCF-7 mitosis and cell division events using the computational filter versus fluorescence in 0 and 10 μM concentrations of cytochalasin B.

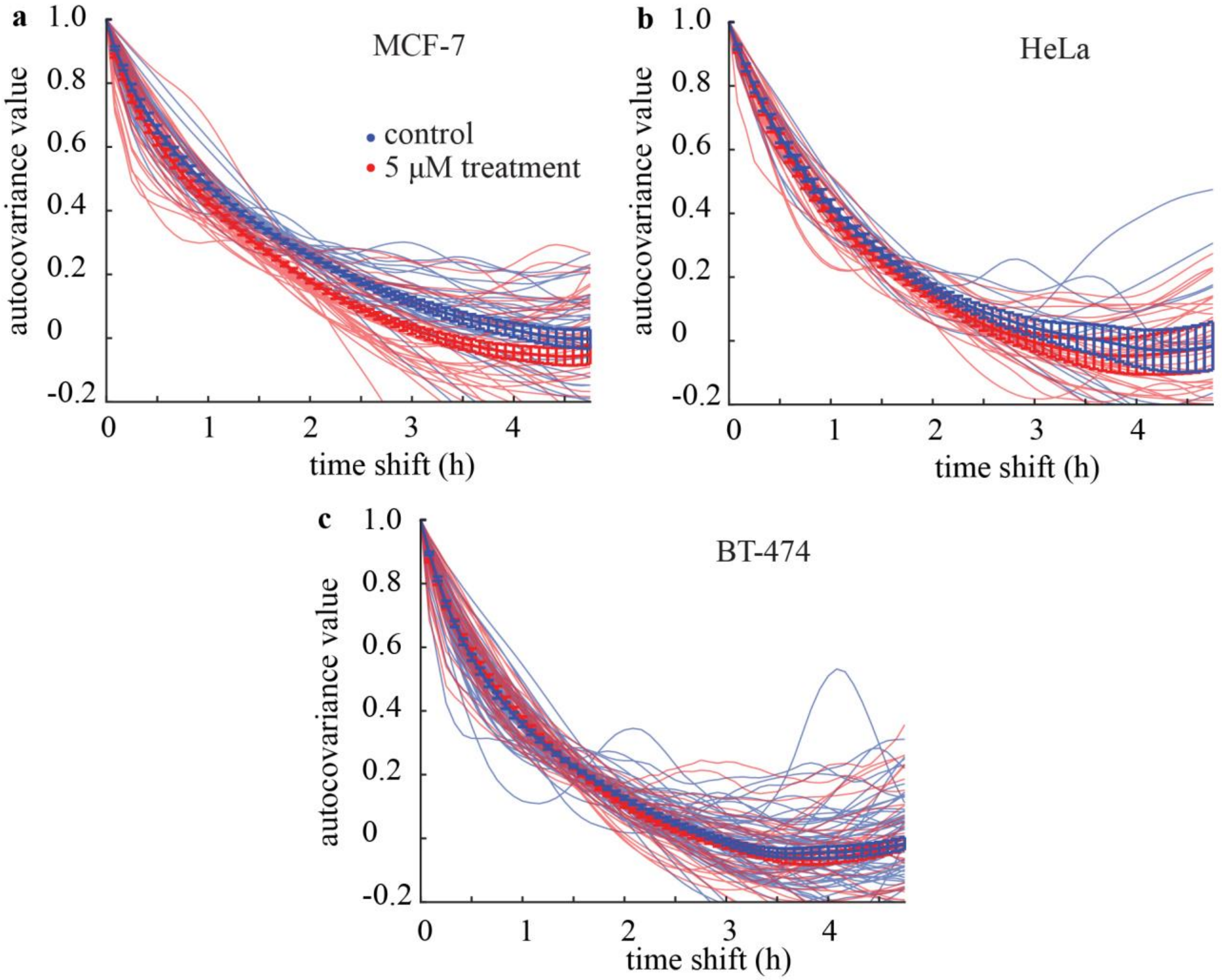

FIGURE S3 The average autocovariance of compliant (aka softer) cells decays more rapidly than for stiffer cells. (**a**) Individual and population averaged autocovariance curves for all 0 (R^2 = 0.99±0.01) and 5 μM (R^2 = 0.99±0.01) cytochalasin B treated MCF-7 cells analyzed. (**b**) Individual and population averaged autocovariance curves for 0 (R^2 = 0.98±0.01) and 5 μM (R^2 = 0.99±0.01) cytochalasin B treated HeLa, and (**c**) 0 (R^2 = 0.98±0.01) and 5 μM (R^2 = 0.99±0.01) cytochalasin B treated BT-474 cells. Error bars represent SEM.

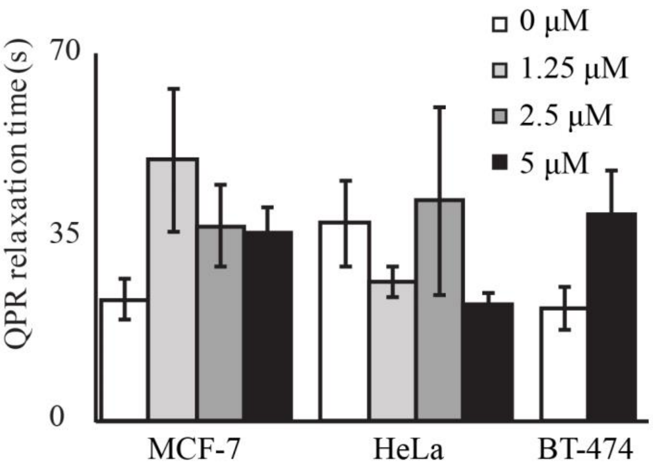

FIGURE S4 Interphase relaxation time calculated from QPR measurements are similar for multiple cell types and drug concentrations. Cells exposed to a range of cytochalasin B doses mainly display similar relaxation times despite changes in stiffness and viscosity. QPR samples were collected at 0 μM (*n*=12), 1.25 μM (*n*=20), 2.5 μM (*n*=14), and 5 μM (*n*=25) for HeLa, at 0 μM (*n*=31), 1.25 μM (*n*=11), 2.5 μM (*n*=22), and 5 μM (*n*=34) for MCF-7, and at 0 μM (*n*=51) and 5 μM (*n*=31) for BT-474 cells. Error bars represent SEM.

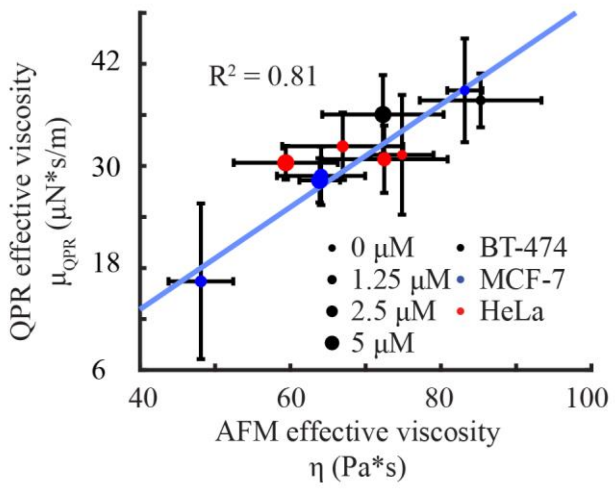

FIGURE S5 QPR predictions for viscosity using a Kelvin-Voight model show reduced correlation with AFM data relative to a Maxwell material model (Fig. 4b, R^2 = 0.89). QPR samples were collected at 0 μM (*n*=12), 1.25 μM (*n*=20), 2.5 μM (*n*=14), and 5 μM (*n*=25) for HeLa, at 0 μM (*n*=31), 1.25 μM (*n*=11), 2.5 μM (*n*=22), and 5 μM (*n*=34) for MCF-7, and at 0 μM (*n*=51) and 5 μM (*n*=31) for BT-474 cells. Error bars represent SEM.

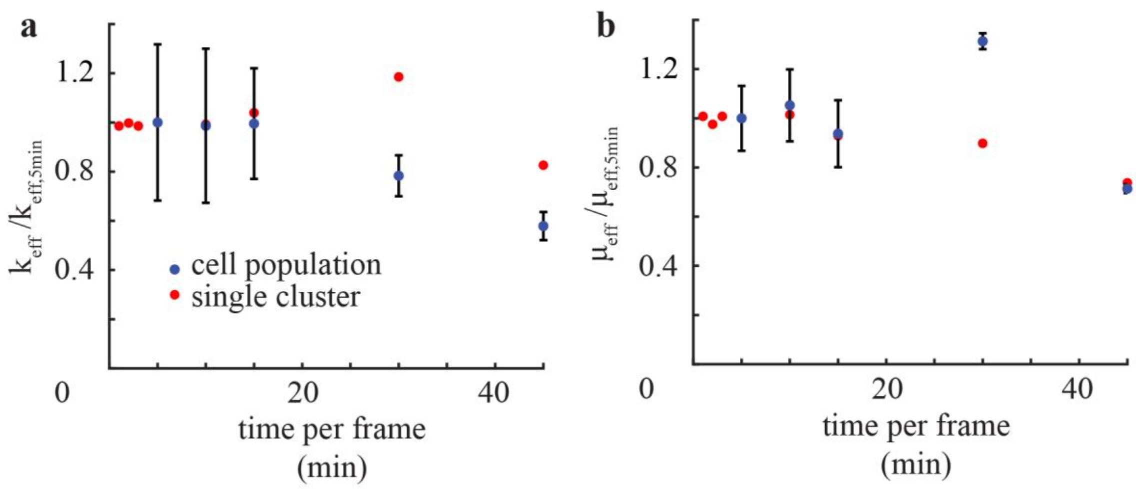

FIGURE S6 Effective viscoelastic modulus of MCF-7 cells at different measurement frequencies remains constant at an imaging rate under 15 minutes per frame. (**a**) Effective stiffness divided by effective stiffness at 5 minutes per frame for a range of QPI measurement frequencies. (**b**) Effective viscosity divided by effective viscosity at 5 minutes per frame for a range of QPI measurement frequencies. Blue represents the population average for analysis of MCF7 cells. A select MCF7 cluster was imaged at higher frequency and is shown in red. Error bars represent SEM.

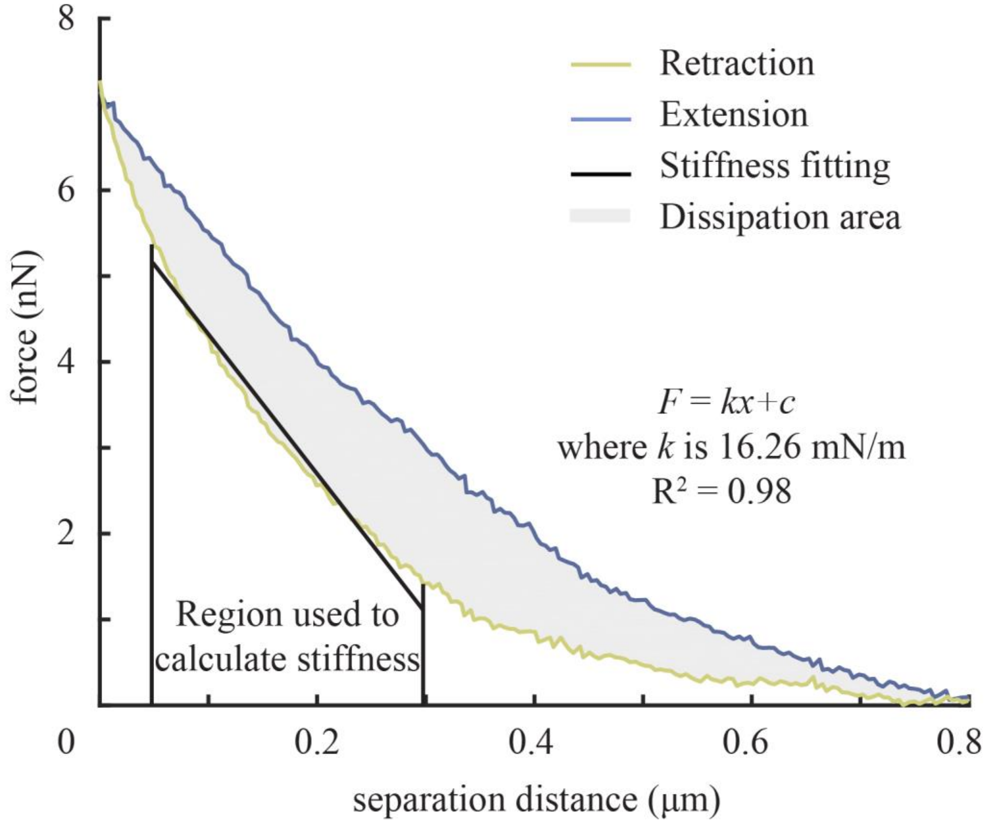

FIGURE S7 Representative AFM stiffness and viscosity measured using best fit of the retraction force curve and area difference between retraction and extend force curve. Data used for effective stiffness from AFM consists of the force data between 20% and 80% of maximum force signal, while viscosity measurements used data over 1 μm distance from contact with cell.

SUPPLEMENTARY UNPROCESSED WESTERN BLOTS

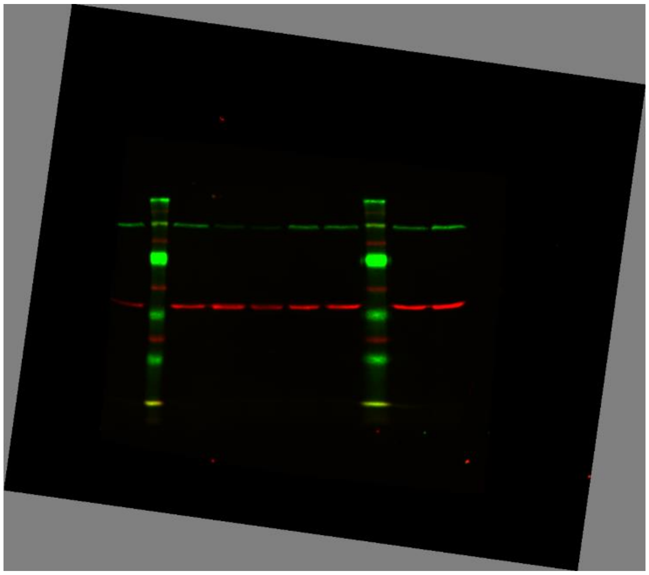

Western blot 1: Unprocessed immunoblot for E-cadherin (green) and β-actin (red) corresponding to Figure 5B.

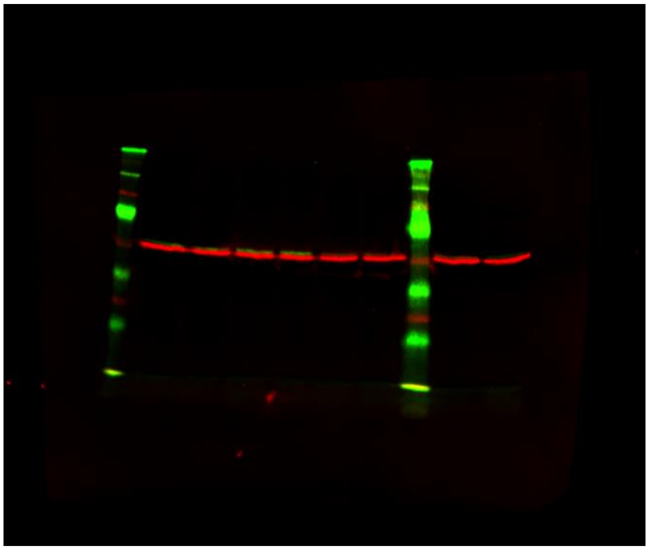

Western blot 2: Unprocessed immunoblot for vimentin (green) and β-tubulin (red) corresponding to Figure 5B.

SUPPLEMENTARY REFERENCES

1 Bendat, J. S. & Piersol, A. G. *Random data : analysis and measurement procedures*. (2013).

2 Lim, C. T., Zhou, E. H. & Quek, S. T. Mechanical models for living cells--a review. *J. Biomech.* **39**, 195-216, doi:10.1016/j.jbiomech.2004.12.008 (2006).

3 Dong, C., Skalak, R., Sung, K. L., Schmid-Schonbein, G. W. & Chien, S. Passive deformation analysis of human leukocytes. *J Biomech Eng* **110**, 27-36 (1988).

4 Davies, H. G. & Wilkins, M. H. Interference microscopy and mass determination. *Nature* **169**, 541 (1952).

5 Barer, R. Interference microscopy and mass determination. *Nature* **169**, 366-367 (1952).

6 Reed, J. *et al.* Rapid, Massively Parallel Single-Cell Drug Response Measurements via Live Cell Interferometry. *Biophys. J.* **101**, 1025-1031, doi:10.1016/j.bpj.2011.07.022 (2011).

7 Mir, M. *et al.* Optical measurement of cycle-dependent cell growth. *Proc. Natl. Acad. Sci. USA* **108**, 13124-13129, doi:10.1073/pnas.1100506108 (2011).
